# Inequalities in access to new medication delivery services among non-communicable disease patients during the COVID-19 pandemic: findings from nationally representative surveys in Thailand

**DOI:** 10.1186/s12939-023-01845-2

**Published:** 2023-02-27

**Authors:** Jiraluck Nontarak, Polathep Vichitkunakorn, Orratai Waleewong

**Affiliations:** 1 Department of Epidemiology, Faculty of Public Health, Mahidol University, Bangkok, Thailand; 2 Department of Family and Preventive Medicine, Faculty of Medicine, Prince of Songkla University, Songkhla, Thailand; 3 International Health Policy Program, Ministry of Public Health, Nonthaburi, Thailand

**Keywords:** Health inequality, Medication delivery services, Medicine reception, COVID-19, Noncommunicable disease (NCDs)

## Abstract

**Background:**

This study describes the inequalities in access to a medication delivery service (MDS) during the COVID-19 pandemic and identifies the social determinants of health-related inequalities among non-communicable disease (NCD) patients.

**Methods:**

Data were obtained from a study on the impact of health behaviours and modifications in health behaviours during the COVID-19 pandemic in the Thai population in 2021. The participants were recruited from Bangkok and all four regions of Thailand. The concentration index was used to examine the inequality among income quintiles, which were standardised by age, sex, living area, job type, health insurance scheme, and education level. Logistic regression was used to examine the associations between socio-demographics and access to regular services and new NCD MDSs, adjusted for age, sex, and other covariates.

**Results:**

Among 1,739 NCD patients, greater income inequalities in accessing regular NCD services and collecting medicines at registered pharmacies during the COVID-19 pandemic were observed, for which the concentration index indicated utilisation inequalities in favour of richer households. In contrast, receiving medicine at primary care centres, by postal delivery, and delivered by village health volunteers were the new NCD MDSs, which favoured less wealthy households. NCD patients living in rural areas were more likely to access new NCD MDSs, compared to those in urban areas (adjusted odds ratio = 2.30; 95% confidence interval [CI]: 1.22–4.34). Significant associations with receiving medicine at hospitals were also observed for the income quintiles. Individuals in the lowest and 2^nd^ lowest income quintiles were more likely to access new MDSs than those in the richest quintiles.

**Conclusions:**

This study highlighted a disproportionate concentration of access to new NCD MDSs during the COVID-19 pandemic in Thailand, which was more concentrated in lower-income groups. The government should further study and integrate MDSs with the highest cost benefits into nationwide regular systems, while addressing systematic barriers to access to these services, such as the lack of shared health data across health facilities and tele pharmacy equipment. This will promote access to public services among patients in the less advantaged groups and reduce the health inequality gap.

## Background

The COVID-19 pandemic has exacerbated multidimensional inequalities worldwide. In countries with existing income inequality and inequalities in access to social protection, including health, education, and other public services, vulnerable populations are most affected by COVID-19 [1]. Many informal and migrant workers have faced job and income losses during this period. COVID-19 has also aggravated health inequalities among patients, including those with noncommunicable diseases (NCDs). NCD patients are more likely to have more severe forms of COVID-19 and subsequent mortality and are affected by the disruption of NCD service delivery [2].

Thailand had national health security preparedness, a strong primary health care, and investment in universal health coverage for early response to COVID-19 [3]. However, one third of the patients who needed medical services were unable to access the required services due to concerns about being infected with COVID-19 [4]. Health services at public hospitals were closed during the peak of the pandemic in mid-2020 owing to a congestion reduction strategy being implemented. Outpatient visits for non-urgent and stable chronic cases were reduced and replaced by teleconsultation services, while NCD medication delivery services (MDSs) were performed by postal delivery. In many areas, medicine for NCD patients was directly delivered by village health volunteers or collected at primary care centres or registered-private community pharmacies [5]. The inequality of accessibility of health care based on social classes, associated with living areas, socioeconomic status, education level, income level, and health insurance types, has already been an issue in Thailand before COVID-19 [6]. These inequalities could also be observed among NCD patients with lower socioeconomic status during the COVID-19 pandemic.

Thailand’s public health agenda faces five major NCDs, namely, hypertension, diabetes mellitus, cardiovascular disease, stroke, and chronic obstructive pulmonary disease (COPD). In 2019, there were 3,528,114 NCD patients nationally, accounting for 5.4% of all patients [7]. This study explores patterns of inequality of access to MDSs among NCD patients during the COVID-19 pandemic by socioeconomic status and living area and determines the social determinants. The results of this study will be useful for public health policies in reducing the gap in NCD MDSs, which will potentially remain regular services in the post-pandemic era.

## Methods

### Study design and setting

We used data from a face-to-face cross-sectional survey, titled ‘A Study of the Impact of Health Behaviours and Modifications in Health Behaviours during the COVID-19 Pandemic in the Thai Population in 2021’. This survey aimed to assess the effects of and changes in health behaviours during the COVID-19 pandemic. Participants aged 15 years or older were surveyed from Bangkok and all four regions of Thailand. In this study, to examine the patterns of inequality in access to MDSs during the COVID-19 pandemic, we recruited NCD patients whose data regarding access to MDSs were complete. Although 7,731 people were initially surveyed, only 1,739 NCD patients were included as they complete data on the access to MDS. The analysis considered the complex survey design, as all estimates were weighted according to the inverse of the probability of being sampled based on the 2021 registered Thai population.

### Measurement of MDSs among NCD patients

Normally, NCD patients collect their medicine at hospitals. The service process includes registration, doctor visits, payment, and medicine collection. During the COVID-19 pandemic, several new NCD MDSs were introduced in Thailand, in addition to the regular services. The survey measured four categories of new MDSs that participants accessed: (1) collecting at primary care centres, a branch of community hospitals, (2) postal delivery, (3) collecting at registered-private community pharmacies, and (4) delivery by village health volunteers (VHV). Participants were allowed to indicate more than one category.

### Measurement of inequality using the concentration index and other statistical analysis methods

The concentration index (CI) was used to examine inequality among the income quintiles, which were standardised by age, sex, living area, job type, health insurance, and education levels, because it has been reported to be suitable for measuring socioeconomic-related health inequality.

The CI was bounded between -1 and 1, which identified whether income quintiles were unequal by different groups of the MDSs. Concentration curves are displayed, which are defined as the area between the concentration curve and the line of equality (the 45-degree line). It was calculated using the following formula

$$C\left(h\backslash y\right) = \frac{2\mathrm{cov}\left({h}_{i},{R}_{i}\right)}{\overline{h}} = \frac{1}{n} \sum_{i=1}^{n}\left\{\frac{{h}_{i}}{h} \left({2R}_{i} -1\right)\right\}$$

In which, *h*_*i*_ denotes the health variable and* R*_*i*_ denotes income [8].

A negative value is displayed when the curve lies above the line of equality, indicating a disproportionate concentration of MDSs among lower-income quintiles (pro-poor), and a positive value when it lies below the line of equality, indicating a disproportionate concentration of MDSs among higher income quintiles (pro-rich). The F-test was used to test the null hypothesis of equality of the index value across sociodemographic groups. The difference in the significance level was set at *p* < 0.05.

The prevalence of access to new NCD MDS was calculated for the overall population stratified by age, sex, area of residence (urban/rural), educational level (no formal education or primary, secondary, and university level), health insurance, and income quintiles. Multiple logistic regression was used to examine the associations between socio-demographics and access to new NCD MDS and was adjusted for age and sex.

All statistical analyses were performed using the Stata version 11.0 software (StataCorp., College Station, TX, USA). The significance level was two-sided, and the values were set at *p* < 0.05.

## Results

### Demographic data and prevalence of medication delivery systems by sociodemographic data

A total of 1,739 participants (51.7% female) were included in the analysis **(**Table 1). The mean age of the participants was 43.5 years (standard deviation; SD: 16.0), and 65.5% of participants lived in rural areas or a de facto area. Of all participants, 34.3% had a primary school level of education, 43.3% were employed in business-type jobs, and 20.4% were employed in labour work. Regarding health insurance, 83% of participants had universal coverage schemes (UC) and approximately 6% had civil servant schemes (CS) and social security schemes (SS). Based on the level of individual monthly income quintile, the 1^st^ quintile had the highest proportion, representing 24.2% of participants, while the 5^th^ quintile showed the lowest proportion (14.7%).

**Table 1:** Demographic data and prevalence of alternative health care by sociodemographic data

Characteristics	Column %	NCD service provision during COVID 19 crisis, row % (95%CI)				
		**Hospitals (*n* = 1,267)**	**New medication delivery services (MDSs)**			
			**Collected at primary health centres (*n* = 615)**	**Delivered by village health volunteer (*n* = 90)**	**Postal delivery (*n* = 30)**	**Collected at registered-private community pharmacies (*n* = 16)**
**Total (row %)**		**72.10 (63.83,79.09)**	**36.41 (27.51,46.35)**	**5.32 (3.46,8.08)**	**1.72 (0.90,3.24)**	**0.94 (0.45,1.94)**
**Sex**						
Male	48.28	73.20 (66.18,80.21)	36.93 (27.27,46.60)	5.55 (2.54,8.57)	1.48 (0.37,2.59)	0.73 (0.11,1.36)
Female	51.72	71.26 (62.39,80.13)	36.06 (26.17,45.95)	5.15 (2.71,7.58)	1.89 (0.51,3.27)	1.09 (0.20,1.98)
**Age group (years)**						
15–24	15.02	82.10 (48.13,116.0)	0.00 (0.00,0.00)	0.00 (0.00,0.00)	0.00 (0.00,0.00)	35.81 (6.27,77.88)
25–39	26.57	76.14 (65.27,87.01)	26.64 (14.86,38.42)	1.21 (-1.35,3.77)	2.04 (0.61,4.68)	1.13 (1.24,3.50)
40–59	40.23	73.55 (65.68,81.43)	36.93 (26.96,46.89)	4.02 (1.89,6.15)	1.55 (0.42,2.67)	0.80 (0.13,1.48)
60 and over	18.18	70.35 (62.12,78.57)	37.23 (27.22,47.23)	6.91 (3.72,10.09)	1.84 (0.25,3.44)	0.79 (0.07,1.52)
**Living Area**						
Rural	65.45	66.46 (58.48,74.44)	44.32 (36.42,52.22)	6.62 (4.01,9.24)	1.65 (0.40,2.89)	0.57 (0.03,1.10)
Urban	34.55	82.50 (72.87,92.14)	21.81 (9.51,34.11)	2.90 (0.40,5.40)	1.85 (0.35,3.35)	1.63 (0.13,3.12)
**Education**						
Non-education and Primary	34.30	69.08 (61.58,76.59)	40.81 (32.15,49.48)	7.06 (4.28,9.84)	1.73 (0.51,2.95)	0.51 (0.04,1.06)
Secondary	45.31	79.48 (71.15,87.82)	28.49 (16.48,40.50)	2.19 (0.43,3.95)	1.66 (0.01,3.33)	1.20 (0.21,2.20)
Bachelor and up	20.39	78.01 (67.52,88.49)	19.65 (6.41,32.90)	1.28 (0.45,3.02)	1.30 (0.47,3.08)	3.90 (1.30,9.10)
**Occupation**						
Employee (government/Private company)	13.69	87.06 (79.61,94.56)	14.74 (2.32,27.15)	1.92 (-1.96,5.81)	1.48 (0.92,3.88)	2.13 (1.01,5.26)
Self-employed (Local business)	43.24	73.66 (64.97,82.34)	34.20 (23.86,44.55)	3.60 (1.16,6.04)	1.95 (0.04,3.95)	1.11 (0.04,21.8)
Agriculture/Labour/informal workers	20.35	66.47 (56.66,76.27)	47.44 (37.25,57.64)	7.59 (4.09,11.08)	0.87 (0.04,1.79)	0.50 (0.26,1.25)
Others	22.72	71.37 (62.54,80.09)	34.26 (24.91,43.61)	7.06 (3.68,10.44)	2.23 (0.46,3.99)	0.77 (0.14,1.69)
**Monthly individual income quintile**						
1^st^ (poorest)	24.23	67.83 (58.73,76.92)	41.81 (32.34,51.28)	8.50 (3.90,13.10)	1.97 (0.40,3.53)	0.36 (0.37,1.10)
2^nd^	17.30	59.82 (49.91,69.73)	46.67 (36.49,56.85)	5.32 (1.84,8.80)	2.96 (0.31,6.22)	0.56 (0.30,1.43)
3^rd^	23.36	78.35 (69.16,87.55)	34.92 (24.38,45.46)	2.57 (0.68,4.45)	1.07 (0.21,2.36)	0.81 (0.06,1.69)
4^th^	20.40	84.16 (76.74,91.57)	20.58 (8.73,32.42)	3.17 (0.60,5.73)	0.46 (0.53,1.46)	1.86 (0.49,4.22)
5^th^ (richest)	14.71	83.44 (77.49,89.39	21.13 (10.63,31.63	3.61 (0.63,6.60)	1.17 (0.87,3.22)	2.54 (0.55,4.54)
**Health insurance**						
UHCS	82.98	70.27 (62.68,77.87)	40.02 (31.16,48.87)	6.19 (3.77,8.60)	1.55 (0.38,2.71)	0.63 (0.07,1.19)
SSS	6.74	89.10 (77.51,100.00)	12.82 (0.10,26.59)	1.68 (-1.95,5.31)	3.07 (0.14,6.00)	0.92 (1.07,2.90)
CSMBS	6.53	87.17 (78.95,95.39)	14.85 (3.01,26.69)	0.88 (-0.90,2.67)	1.84 (0.88,4.55)	1.82 (0.76,4.41)
Others	3.75	54.08 (37.49,72.11)	36.05 (17.65,55.35)	0.00 (0.00,0.00)	2.89 (1.22,7.00)	6.48 (0.14,12.81)

*NCD* Non-communicable disease, *MDS* Medication delivery service, *CI* Confidence interval, *UHCS* Universal health coverage scheme, *SSS* Social security scheme, *CSMBS* Civil servant medical benefit scheme. *n* = 1,739 with a total of 2,018 NCD service provisions

Overall, 72.1% of NCD patients still had access to regular services or received medicine at hospitals, while 36.4% of them received medicine at primary health centres, 5.3% received medicine at home from deliveries by VHV, and 0.9% collected medicine at registered pharmacies. Among those receiving medicine from pharmacies, 35.8% were individuals aged 15–24 years. Among those collecting the medicine at primary health centres, 44% lived in rural areas and 22% lived in urban areas. Regarding income levels, approximately 80% of individuals in the 4^th^ and 5^th^ quintiles received medicine at hospitals. Individuals in the lower quintiles (1^st^ and 2^nd^ quintiles) had a higher proportion of patients receiving medicine at primary health centres. In contrast, people in the 5^th^ quintile had the highest proportion of patients receiving medicine at registered pharmacies.

### Concentration index curves of health inequality by income quintiles

**Fig. 1:**
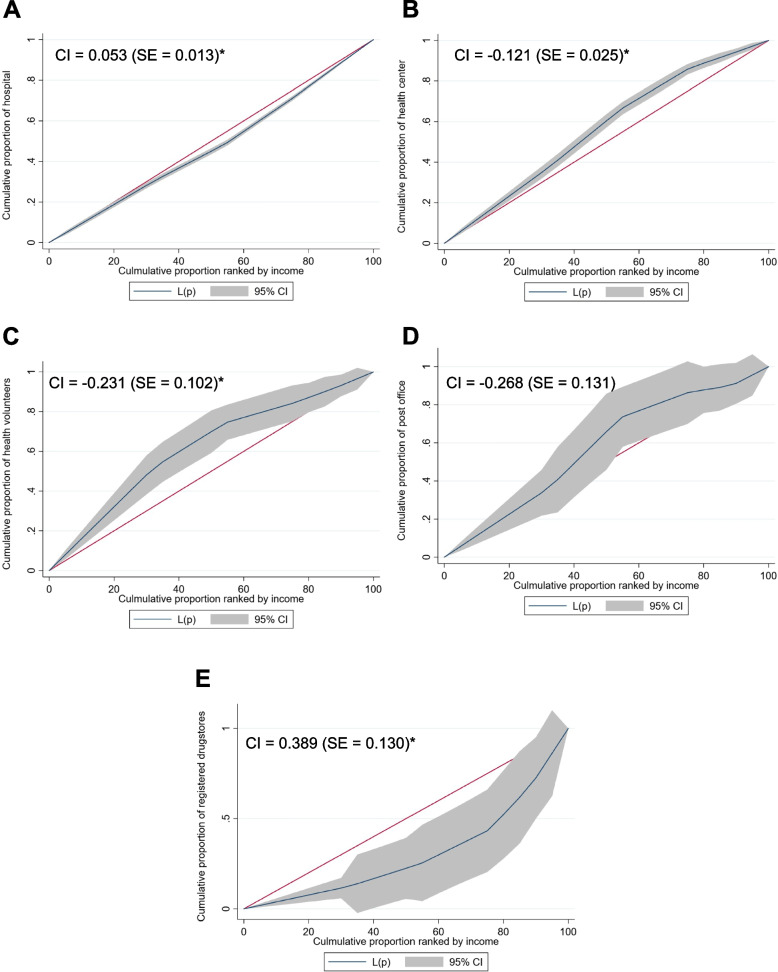
Concentration curve of medical delivery systems during COVID-19 pandemic by income quintile. A is the concentration curve of the hospitals, B is the concentration curve of the health centre, C is the concentration curve of the health volunteers, D is the concentration curve of the post office, and E is the concentration curve of the registered drugstores. Note: * *p*-value < 0.05, standardised for age, sex, living area, job type, health insurance, and education levels. CI: concentration index; SE: standard error

There is some evidence suggesting that concentration indices for accessing new NCD MDSs during the COVID-19 pandemic are more sensitive to the level of income in this population. Figure 1 shows the substantial and significant differences between the CI for four categories of new NCD MDSs among NCD patients and income quintiles. Regarding regular services or receiving medicine at hospitals, the CI indicated statistically significant inequality in favour of richer households (CI index = 0.053 [standard error (SE) = 0.013]). In addition, the inequality was greater for receiving medicine at registered pharmacies among different income levels, for which the CI indicated inequality in utilisation in favour of richer households. In contrast, receiving medicine at primary health centres, logistics services, and VHV were the MDSs in favour of less wealthy households.

### Inequality of medication delivery systems compared across population groups

The estimates of all inequality indices compared across the population groups are shown in Table 2. There was a significant concentration of those receiving medicine at hospitals among those receiving higher income in both rural and urban locations (F-stat = 11.509, *p*-value < 0.001). Regarding occupation types, receiving medicine at hospitals showed a significant concentration in favour of higher income groups among different occupation types (F-stat = 5.463, *p*-value < 0.001). However, receiving medicine at primary health centres indicated inequality in support of lower-income groups among different occupation types (F-stat = 3.363, *p* = 0.018).

**Table 2:** Tests of the null of equality of index values (income quintiles and across population groups)

NCD services	Insurance groups (F-stat)	Living area (F-test)	Education level (F-test)	Occupation (F-test)
**Hospitals**	F-stat = 0.975 *p*-value = 0.404	F-stat = 11.508 *p*-value = 0.001*	F-stat = 0.464 *p*-value = 0.629	F-stat = 5.463 *p*-value = 0.001*
**New NCD MDS**	F-stat = 0.866 *p*-value = 0.4578	F-stat = 2.966 *p*-value = 0.085	F-stat = 0.240 *p*-value = 0.786	F-stat = 2.6675 *p*-value = 0.046
Primary care centre	F-stat = 1.077 *p*-value = 0.358	F-stat = 0.375 *p*-value = 0.847	F-stat = 0.963 *p*-value = 0.382	F-stat = 3.363 *p*-value = 0.018*
Village Health volunteers	F-stat = 2.0215 *p*-value = 0.133	F-stat = 0.430 *p*-value = 0.512	F-stat = 0.471 *p*-value = 0.624	F-stat = 1.121 *p*-value = 0.339
Postal delivery	F-stat = 0.948 *p*-value = 0.417	F-stat = 0.550 *p*-value = 0.459	F-stat = 0.940 *p*-value = 0.391	F-stat = 1.651 *p*-value = 0.176
Registered pharmacies	F-stat = 0.2316 *p*-value = 0.874	F-stat = 0.296 *p*-value = 0.587	F-stat = 0.157 *p*-value = 0.855	F-stat = 2.652 *p*-value = 0.047*

*NCD* Non-communicable disease, *MDS* Medication delivery service. *n* = 1,739 with a total of 2,018 NCD service provisions

### Association between socioeconomic factors and access to new NCD MDSs among NCD patients during the COVID-19 pandemic (*n* = 1,739).

The adjusted odds ratios (AORs) of receiving medicine at hospitals and other sociodemographic variables are shown in Table 3. Adjusted for other variables, participants living in rural areas were 2.30 (95% confidence interval [CI]: 1.22–4.34) times more likely to access new NCD MDSs than those living in urban areas.

**Table 3:** Socioeconomic factors associated with access to new NCD medication delivery services compared to regular services

**Characteristics**	**Unadjusted OR**	**95% CI**		**Adjusted OR**	**95% CI**	
		**LL**	**UL**		**LL**	**UL**
**Sex**						
Male	1.00			1.00		
Female	1.10	0.87	1.40	1.05	0.79	1.41
**Age group (years)**						
15–24	1.00			1.00		
25–39	1.44	0.12	16.95	2.43	0.20	29.03
40–59	1.61	0.15	17.44	2.04	0.18	23.69
60 and over	1.86	0.17	19.82	1.77	0.16	19.93
**Living Area**						
Urban	1.00			1.00		
Rural	2.46	1.18	5.12	2.30*	1.22	4.34
**Monthly individual income quintile**						
5th (richest)	1.00			1.00		
2nd	3.60	2.16	5.99	3.00*	1.59	5.65
3rd	1.51	0.86	2.64	1.30	0.68	2.47
4th	0.98	0.56	1.73	0.98	0.57	1.68
1st (poorest)	2.57	1.49	4.44	2.11*	1.09	4.09
**Education**						
Non-education and Primary	1.00			1.00		
Secondary	0.58	0.43	0.79	0.72*	0.55	0.96
Bachelor and higher	0.63	0.37	1.09	1.28	0.67	2.46
**Health insurance**						
UC	1.00			1.00		
SS	0.27	0.10	0.77	0.45	0.17	1.19
CS	0.33	0.15	0.72	0.42	0.17	1.03
Others	1.94	1.06	3.54	2.59*	1.45	4.62
**Occupation**						
Employee (government/Private company)	1.00			1.00		
Self-employed (Local business)	2.73	1.50	4.99	1.12	0.56	2.23
Agriculture/Labour/informal workers	3.89	1.91	7.92	1.30	0.64	2.63
Others	3.05	1.61	5.78	1.13	0.63	2.03

*NCD* Non-communicable disease, *OR* Odds ratio, *CI* Confidence interval, *UC* Universal coverage scheme, *SS* Social security scheme, *CS* Civil servant scheme

Significant associations were observed between access to new NCD MDSs and income quintiles. Participants in the 2^nd^ income quintile were 3.00 (95% CI: 1.59–5.65) times more likely to access new NCD MDSs compared to those in the 5^th^ quintile (richest quintile). Furthermore, individuals in the poorest group were 2.11 (95% CI: 1.09–4.09) times more likely to access new NCD MDSs than those in the richest group. In contrast, participants with other health insurance (private insurance or out-of-pocket) were more likely to receive medicine from alternative delivery systems than participants with the Universal Health Coverage Scheme (UHCS; AOR = 2.59, 95% CI: 1.45–4.62).

## Discussions

Health inequalities during the COVID-19 pandemic could be explained by various factors, such as infection rates, disruption of NCD services, and health system capacity in response to crises, which vary across areas. This study found health inequalities regarding differences in access to NCD MDSs by income group during the COVID-19 pandemic in Thailand. Richer households were more likely to receive medicine at hospitals and registered pharmacies, whereas poorer households were more likely to receive medicine at primary health centres and postal delivery. Moreover, there was a significant difference in access to NCD MDSs between individuals living in both urban and rural areas, as well as by occupation type (governors, local businesses, labour forces, and others). The determinants of access to new NCD MDSs were living area, income quintiles, education level, and health insurance schemes.

This study applied monthly income as the main determinant of the four new NCD MDS categories. There were significant differences in monthly income levels between those living in urban and rural areas, which affected access to NCD MDSs.

Individuals living in urban areas tend to still use regular services or receive medicine at hospitals, whereas those living in rural areas tend to receive medicine at primary health centres. This follows the health care utilisation pattern in normal times in Thailand, which is generally based on the availability and geographical accessibility of health facility types. In this study, individuals living in urban areas were less likely to use new MDSs than those living in rural areas. This may indicate that health services in urban areas, which have a higher capacity to respond to crises, were less disruptive than those in rural areas [9]. Concurrently, urban patients may not be affected by the lockdown policy and be able to travel to hospitals owing to the greater availability of public transportation or a shorter distance from home to hospitals in urban areas [10, 11, 11–16]. However, new NCD MDSs, which are more accessible to people living in rural areas, provide more benefits and convenience to patients regarding time and travel costs [17]. The new NCD MDSs are implied as substituted goods to replace the regular MDSs [18]. In this study, the innovative practices seem to be practical in sustaining the service while it satisfies NCD patients in rural areas.

Similarly, patients in the more advantaged groups, such as the higher income group, with Civil Servant Medical Benefit Scheme (CSMBS), and secured jobs (government officers or self-employed), were more likely to receive medicine from hospital services than other new MDSs. In contrast, patients in the less advantaged group, such as the lower-income group, being informal workers, tend to receive medicine at primary care centres. Additionally, individuals with the UHCS were more likely to receive medicine from new MDSs than those with the CSMBS and Social Security Scheme (SSS). A recent national survey in 2019 showed that the disparity in unmet needs across the three insurance schemes was associated with the disparity in socioeconomic status. The members of UHCS occupy a lower proportion in the richest quintile (14.9%), while 53.6% of CSMBS members belong to the richest quintile. This resulted in CSMBS members having the lowest total unmet need compared to the other two insurance schemes. Furthermore, the richer wealth quintiles had lower total unmet needs than the poorer quintiles [19]. Focusing on the reasons for unmet outpatient needs, long waiting times, no time to seek care, and inconvenient transportation were among the main reasons, while the cost of treatment was not an issue [19]. Thus, the new NCD MDSs, such as telepharmacy and receiving medicine at registered pharmacies, have revealed their practicality and potential in addressing the common unmet needs among the poor, the members of UHCS, and other health insurance schemes. The proposal on the implementation of NCD medicines at pharmacies is also supported by a previous study which revealed its cost as well as benefits and gained patient satisfaction owing to a shorter waiting time and reduced travelling costs [20].

This study had some limitations, particularly regarding the ability to interpret the findings due to the lack of relevant variables. First, the questionnaire from this survey project did not measure the reasons or barriers that may reduce access to health services during the pandemic, such as physical distance from the patient’s house to their health care facilities. Consequently, we were unable to clearly interpret which social determinants influenced the inequality in access to services. Second, data regarding NCD service disruption or data provided by health facilities in the areas of study were lacking. Third, the information on ‘asset income’ was not shown in the survey; therefore, only the ‘monthly income’ from salary was calculated for the income quintile, which may be a less sensitive indicator of socioeconomic status [21]. Finally, there were some limitations regarding small sample sizes in some categories of new NCD MDSs. Thus, this study may not significantly indicate the statistical differences.

## Conclusions

This study highlighted a disproportionate concentration of the use of new NCD MDSs, which was more concentrated in the low-income group. Owing to the relative benefits gained, it is more likely that the Ministry of Public Health will continue implementing the congestion reduction strategy at health facilities after the pandemic and embedding new NCD MDSs into existing systems, such as collecting medicine at private pharmacies or primary health centres, including telepharmacy. Therefore, the government should further study and integrate MDSs with the highest cost benefits into regular systems nationally. Furthermore, the government should actively identify lower-income individuals who have less access to new services and provide them with specific services or support to overcome systematic barriers, such as equipment used for teleconsultation services. Moreover, the government should intensively strengthen a shared health big data system which allows health facilities to access patient profiles and history of treatment, provide care to the patients regardless of their location, and should not be limited to registered health services. This will promote access to public services among patients in the less advantaged groups and reduce the health inequality gap.

## Data Availability

The datasets used and analysed during the current study are available from the corresponding author on reasonable request.

## Abbreviations

MDS: Medication delivery service
NCD: Non-communicable disease
CI: Confidence interval
COPD: Chronic obstructive pulmonary disease
AH: Area health
VHV: Village health volunteers
CI: Concentration index
UC: Universal coverage schemes
CS: Civil servant schemes
SS: Social security schemes
SE: Standard error
AORs: Adjusted odds ratios
UHCS: Universal health coverage scheme
CSMBS: Civil servant medical benefit scheme
SSS: Social security scheme
OR: Odds ratio
